# Diabetes-Related Healthcare Services in Nepal—A Qualitative Exploration of Healthcare Professionals’ Opinions

**DOI:** 10.3390/pharmacy8030131

**Published:** 2020-07-29

**Authors:** Sujata Sapkota, Jo-anne E. Brien, Parisa Aslani

**Affiliations:** 1 School of Pharmacy, Faculty of Medicine and Health, The University of Sydney, Camperdown, NSW 2006, Australia; jo-anne.brien@sydney.edu.au; 2 Manmohan Memorial Institute of Health Sciences, Soalteemode, Kathmandu 44614, Nepal; 3 St. Vincent’s Hospital Clinical School, The University of New South Wales, Darlinghurst, NSW 2010, Australia

**Keywords:** type 2 diabetes, diabetes care, healthcare professionals, qualitative study, Nepal

## Abstract

Healthcare professionals’ level of engagement in diabetes care and their perceptions of challenges to effective diabetes care are key indicators impacting diabetes management. This study investigated diabetes-related healthcare services provided in Nepal, and explored healthcare professionals’ opinions of the barriers to, and strategies for, effective diabetes care. In-depth face-to-face interviews were conducted with thirty healthcare professionals providing healthcare or medication-related services to patients with type 2 diabetes within Kathmandu Valley. Interviews were audio-recorded, transcribed verbatim and thematically analysed. Participants were physicians, dieticians, nurses and pharmacy staff. Diabetes care services varied between healthcare institutions, between healthcare professionals and between patients, with the overall patient-care model reported as sub-optimal. Diabetes related services were mostly limited to physician-patient consultations. Only a few hospitals or clinics provided additional diabetes education classes, and individual dietician or nurse consultations. Limited collaboration, large patient-load and workforce shortages (particularly lack of diabetes educators) were reported as major issues affecting diabetes care. Regulatory measures to address healthcare system barriers were identified as potential facilitators for effective diabetes management. Whilst the findings are specific to Nepal, there are lessons to be learnt for other healthcare settings as the fundamental barriers to optimal diabetes care appear to be similar worldwide.

## 1. Introduction

Nepal, like most countries in the world, is facing an increasing burden of type 2 diabetes (T2D), particularly in urban areas [1,2]. Diabetes is a complex chronic condition, associated with debilitating complications [3]. Lifestyle management, including a balanced healthy diet and adequate physical exercise, together with anti-diabetic medications are the basis of diabetes treatment [4]. 

Considering the nature of diabetes and its management, different healthcare professionals (HCPs) have unique and significant roles to play in diabetes care. The HCPs most commonly involved in day-to-day diabetes care include the general (or family) medical practitioner/ physician, endocrinologist, dietician, diabetes educator, nurse, pharmacist and podiatrist. Other specialists (such as, cardiologist, ophthalmologist and nephrologist) are also involved, particularly in assessing and managing the complications of diabetes. For effective treatment outcomes, it is advocated that these HCPs work in collaboration with patients and their families to provide an integrated care focused on patient needs and to ensure that the patient receives pertinent information, counselling and support throughout the diabetes journey [4]. 

The healthcare system itself can have an impact on diabetes care. Studies have emphasised the roles of HCPs and healthcare systems in effective diabetes management [5]. For example, HCPs’ knowledge/skills, support, time, and institutional and healthcare limitations are known to impact diabetes care [6]. Effective management of the increasing incidence of chronic diseases, such as diabetes is challenging even for high-income countries with modern and advanced health facilities [7]. Diabetes prevention and management in low-income countries face greater challenges [7,8].

In Nepal, diabetes has been associated with ‘catastrophic health expenditure’ [9]. Whilst the prevalence of diabetes and its complications are rapidly increasing in Nepal, limited healthcare facilities, high costs associated with treatment, inadequate awareness about diabetes in patients and lack of specific guidelines for diabetes management all pose challenges to effective diabetes management [10]. Additionally, diabetes care in Nepal can be influenced by the utilisation of healthcare by the Nepalese who have unique socio-cultural characteristics and preferences for indigenous healthcare practices [11,12,13,14]. A meticulous investigation of diabetes care delivery processes in Nepal and the factors impacting the healthcare delivery processes at the healthcare system and HCP level is expected to elucidate the gaps in the care processes and to create the foundation for concrete future planning and strategies to optimise diabetes care in Nepal. Although international studies have explored HCPs’ views on the management of T2D [15,16,17], studies exploring HCPs’ experiences, roles and opinions about diabetes management in Nepal, a resource limited country, are limited. HCPs working in the Nepalese healthcare system and involved in diabetes care are the most appropriate professionals to assess healthcare services and healthcare-related issues in diabetes management.

This study, therefore, aimed to investigate diabetes-related healthcare services provided in Nepal from the experiences of the Nepalese HCPs involved in diabetes care, and to explore their opinions of the barriers to, and strategies for, effective diabetes care delivery.

## 2. Materials and Methods

### 2.1. Study Design

Qualitative research was the approach used, as it was the most appropriate method to address the research objectives. Qualitative research is highly valuable as the first line of investigation [18], and is preferred when there is little pre-existing knowledge and limited data or explanations; and when detailed exploration about the topic of interest is desired [19,20]. Furthermore, qualitative research techniques have the capacity to provide complex textual descriptions of peoples’ experiences of a given research issue and are particularly effective in obtaining information specific to socio-cultural context and in effectively identifying information about behaviours and practices [21,22]. Semi-structured in-depth interviews were used as they allow for a structured interview plan and questioning framework while also maintaining the exploratory and conversational nature of the method [23]. Interviews are also a more appropriate approach when participants are busy HCPs who cannot all attend at the same time for a focus group discussion.

Ethical approval to conduct the study was obtained from Nepal Health Research Council, Kathmandu, Nepal (Approval Registration number: 86/2015). 

### 2.2. Sample

Based on previous research [12,24] in Nepal, HCPs providing traditional (Ayurvedic) medicine as well as HCPs providing modern medicine and involved in managing patients with T2D in Kathmandu Valley, were identified and recruited using multiple strategies. HCPs were identified through:

- Web-pages of different hospitals and healthcare centres.
- Specific hospitals, clinics and pharmacies in Kathmandu Valley providing services to people with diabetes.
- ‘Snowballing’: Recruited HCPs were requested to contact other HCPs who may be willing to participate in the study, and to ask those HCPs to contact the researcher for participation. Similarly, the recruited HCPs were requested to provide the public contact details of other HCPs who may be interested in the study, for the researchers to contact them.

HCPs were contacted through the available contact details (email or phone) or in-person, or through a letter of invitation delivered to them via their healthcare organisations. Participants were provided with the participant information statement which outlined the study and the study process. Follow-up was conducted as appropriate either by email, telephone or in person to organise a time and venue for the interview. 

### 2.3. Data Collection

HCPs who agreed to participate and provided written consent were interviewed face-to-face using a pre-designed and validated interview guide consisting of open-ended questions that explored broad issues related to diabetes care in Nepal (Table 1).

A detailed interview guide was prepared by the research team based on the research objectives. The protocol was also guided by the findings of the interviews with the patients [12,24,25,26]. Additionally, opinions of two other researchers, who were experts in qualitative research, public health and intervention studies (focussing on chronic diseases), were sought.

This paper presents the findings related to HCPs’ reports of the services provided to patients with T2D in their hospitals and clinics, their roles in the management of patients, and their experiences and opinions about the healthcare system barriers to effective delivery of diabetes care to patients. 

The interviews were conducted by the lead author at the HCPs’ institutions, except for two interviews which were conducted in cafes at the request of the HCPs. While three HCPs responded to the questions in English, which consequently became the primary language in these interviews; the primary language in other interviews was Nepali. The interviews lasted from 23 to 61 minutes. Interviews were audio-recorded (except one, where the HCP requested note-taking only) and field notes written after each interview. Demographic data for each HCP were collected. Interviews were conducted until data saturation and redundancy was reached [27]. Data saturation was sought overall rather than between HCP specialties. Data saturation was reached at the 26th interview; however, four more interviews were conducted to confirm that no new themes emerged. 

### 2.4. Data Analysis

The audio-recorded interviews were transcribed verbatim; and data were analysed using thematic analysis [28]. Analysis of qualitative data can involve methods related to a specific theoretical or epistemological position, for example, grounded theory and phenomenological analysis, or methods that are typically independent of theory, offering theoretical freedom and flexibility in analysis, such as thematic analysis [28]. Thematic analysis is recognised as an independent, reliable, and useful research tool that is able to generate a rich and detailed, yet complex, account of qualitative data [28,29]. In addition to providing a rigorous, flexible approach to produce an insightful analysis of the research questions [28], it also complemented the study aims, by facilitating the exploratory analysis of the diabetes management situation in Nepal, and allowing the themes to be ’described, compared and related‘ between different practitioners [30]. 

All transcripts, including the notes taken during one interview, were carefully read line by line, and an initial label code was applied as an interpretation of the lines, statements or passages, as appropriate [31]. Interviews conducted in English were analysed independently by two researchers and consensus regarding themes and Sub-Themes were reached. Open coding using an inductive approach was used [31]. The initial codes were then categorised, organised or grouped into themes and Sub-Themes [28,31,32]. Findings were discussed to refine and clarify emerging themes within the data until a consensus was achieved. NVivo10 Software (QSR International (Americas) Inc, Burlington, Massachusetts, MA, USA) was used to manage and code data. The field notes were examined and used to support the analysis and interpretation of the data. 

As the interviews were conducted with HCPs of multiple specialties, data were evaluated between individual HCPs, as well as within and across the specialties to explore similarities, differences and/or any unique features associated with the specialty. The cross-comparison method was also applied throughout the data analysis process. 

## 3. Results

### 3.1. Participant Demographics

A total of 30 HCPs were interviewed (Table 2). The participants were recruited from 13 hospitals and clinics providing modern (allopathic) healthcare services; and six clinics providing traditional (Ayurvedic) services. Three professionals providing pharmacy services were independent owners of three community pharmacies (Table 2). Diabetes educators could not be recruited for interviews as we could not identify any healthcare professionals with formal diabetes educator certification or who identified themselves as ‘Diabetes Educators’.

### 3.2. Interpreted Themes

Four themes emerged from the qualitative analysis of the in-depth interviews (Table 3). 

#### 3.2.1. Theme 1: Delivering Care for Patients with Diabetes 

**Figure 1:**
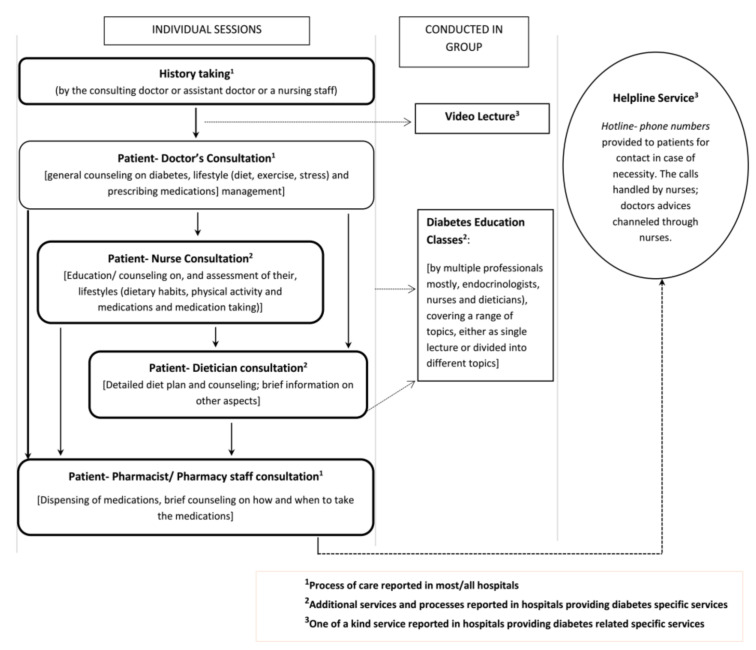
Reported healthcare consultation and education opportunities available for diabetes patients in Nepal.

This theme describes the reported services delivered to patients with diabetes. The healthcare services provided to patients with T2D in Nepal were reported to vary between hospitals, between clinics, and between hospitals and clinics, as well as between care providers (Figure 1). The services ranged from general ‘check-up’ and physician-patient consultation services in most hospitals, to diabetes specific services in a few hospitals. A few hospitals had a separate ’Endocrinology’ unit; and a few were entirely focused on specialist services for patients with ’Diabetic and Endocrine’ disorders. Overall, diabetes-specific services were mostly provided by these specialist hospitals and clinics. 

The diabetes specific services mostly included education (classes, video lectures), in-house dietician consultation and specific diabetes counselling from nurses, in addition to regular physician-patient consultations. The physicians who provided services in settings which offered diabetes classes or additional individual counselling opportunities for patients reported that they encouraged patients to attend the classes and referred patients for individual counselling with nurses or dieticians. Educational video lectures were used in one hospital, where the endocrinologist asked the patients to watch the lecture before their consultation. Similarly, a unique telephone *’Helpline System*’ where patients could contact, or were contacted, to report any issues was offered by one ’*care centre*‘(P18, Physician). Nurses handled the telephone calls; and depending upon the nature of the reports, they offered counselling either themselves or after consultation with physicians. Endocrinologists and/or diabetologists were involved in conceptualising, supervising and facilitating these services. 

Laboratory blood glucose monitoring services were available in most healthcare settings where interviews were conducted. Additionally, a number of private laboratory services in Kathmandu (outside hospitals and healthcare centres) were accessible to patients. Two pharmacists working in a community setting also offered laboratory blood monitoring services directly to patients. 

Participants did not report a standardised or consistent approach to diabetes care by healthcare professionals, nor a cohesive or continuity of care that would be expected for a chronic condition such as diabetes, which involves a range of healthcare professionals for optimal patient care. It appeared that, in the majority of cases, the participants were working in silos, with very limited interprofessional collaboration or indeed, communication. Whilst a broad range of services were noted, services appeared to be primarily centred around patient–physician consultations. 

#### 3.2.2. Theme 2: Healthcare Professionals Partnering for Wholistic Care in Diabetes Management

The roles of participating HCPs in diabetes management varied according to their specialties. However, all reported that educating patients about diabetes related issues was one of their key roles, and an important role in the wholistic care of their patients.

Physicians (including specialists), dieticians, nurses and pharmacy staff were commonly involved in providing healthcare and medication-related services to patients with diabetes in Nepal. Some Ayurvedic physicians, practicing traditional (Ayurvedic) methods of care, also attended to the healthcare needs of patients with T2D.

##### Physicians’ Role

Overall, it was reported that physicians provided the majority of counselling and treatments. Their role in educating patients about diabetes and treatment (lifestyle and medications) formed a fundamental part of their consultations. Physicians perceived themselves as ’*a physician and an educator*‘(P3, Physician; P8, Physician) and stated that ’*making patients understand (the disease and treatment)*’ (P14, Physician) was their role. They also referred patients for dietary consultations, for consultations with diabetes specialists (by physicians or internal medicine specialists), or to other specialists, such as nephrologists and ophthalmologists, for complications management. 

##### Nurses’ Role

The participating nurses reported that they were involved in providing assistance in assessing patients’ lifestyle and medication taking behaviour and providing advice, either through individual or group patient education sessions. They worked in close collaboration with endocrinologists and diabetologists to facilitate patients’ diabetes care. 

##### Dieticians’ Role

Six of the 13 hospitals in this study reported to have in-house dieticians. Dieticians planned and managed patients’ diet and provided dietary counselling and education. Some reported providing other services, such as advice on exercise, in their consultations. An individual session with a dietician entailed evaluation of the patient’s existing dietary practices and physiological parameters (height, weight, BMI), and designing an individualised dietary plan based on the patient’s energy (calorie) requirements. Most patients seen by the dieticians had been referred by physicians, however, in recent years, dieticians reported that more patients were seeking dieticians of their own accord. In healthcare centres with dieticians on site, physicians tended to refer patients for dietary consultations. In centres without a dietician, physicians or sometimes nurses would provide dietary advice. 

##### Pharmacists/Pharmacy Staffs’ Role 

Dispensing medications and counselling on how and when to take the medications was the main reported role of pharmacy staff (pharmacists and pharmacy professionals) in diabetes care. Pharmacy professionals, include pharmacy staff other than formally qualified pharmacists and pharmacy assistants, who can engage in pharmacy services in Nepal, with basic training in medication dispensing, under the provision set by Drug Act 1978 AD of Nepal. A few pharmacy staff stated that patients coming for a refill generally knew how to take their medications and did not require counselling; their role, in such cases, was limited to supplying medications. Nonetheless, the pharmacy staff reported that they provided information about diabetes or medications to the patients who sought information from them or were *‘willing to listen’* (P29, Pharmacist).

The type and amount of counselling from a pharmacy staff largely depended on patients’ interest and attitude towards the service provider. The pharmacy staff reported that not everyone was willing to receive information from pharmacy staff; and ‘*forcing information*’ was perceived ‘*pointless*’ (P30, Pharmacy Staff). They reported that most of the times, patients were rather impatient to collect medications and leave; and were not always appreciative of the advice given to them. A hospital pharmacist stated that supplying information voluntarily was interpreted as, pharmacist trying to ‘*sell medications*’.

*‘Here we speak with limitations; if we speak more the patient is irritated. Yes, patients get irritated. If we start teaching them a lot of thing, what he (the patient) feels is that he (the pharmacist) is saying all these to sell his medications. He thinks that we are acting as if we know everything. You shouldn’t speak unless they ask… that will wreck everything!’. (P29, Pharmacist)*

Lack of awareness amongst the public that ‘*medical shops are owned and run by qualified professionals*’ (P24, Pharmacist) was reported as a reason for peoples’ lack of trust and expectation of professional services from pharmacies. Nonetheless, a few participants working in community pharmacies reported that people (yet undiagnosed) approached them with diabetes-related symptoms, seeking advice and care; their role in such cases involved guiding diagnosis (or conducting laboratory tests that some pharmacies offered) based on reported symptoms and referral along with advice and counselling. 

Whilst the pharmacy staff interviewed recognised effective patient education and counselling as a key to optimising diabetes management in patients, they acknowledged that they were not providing adequate counselling or services to the patients with diabetes. In hospital pharmacies, a pharmacist reported that their counselling role was not distinctly classified; they also felt that the hospital administrators were not appreciative of their ’unique’ role.

*‘Now one thing that you have been employed here is to sell medications. If you start doing other things, there is so much crowd in the pharmacy, the director will come and reprimand you’. (P29, Pharmacist)*

A further reason for the self-perceived lack of quality services being offered from hospital pharmacies was insufficient pharmacy workforce to deal with the patient-load during ’peak hour’. None of the participating pharmacy staff reported to work in close collaboration with physicians to assist in patients’ diabetes management. 

##### Traditional Medication Prescribers’ Role

Physicians practicing traditional (Ayurvedic) medicine reported that a number of patients with diabetes visited them seeking treatment using traditional (herbal or Ayurvedic) methods. Similar to the physicians involved in modern medicine, they delivered counselling on lifestyle management and provided treatment (in the form of herbs and Ayurvedic medications). They reported that patients’ lifestyle and daily routine held a great significance in Ayurveda, therefore understanding and managing patients’ lifestyle was important, and a significant component of their role in diabetes management. It was reported that while Ayurvedic and herbal medications helped in lowering blood glucose to a certain extent, patients, particularly with uncontrolled diabetes and on insulin, had to be referred for modern (allopathic) medications, as Ayurvedic medications alone were not sufficient for effective diabetes control. In such cases patients were either encouraged to seek help from allopathic prescribers (if they had not already done so), or to continue their allopathic medications and consultations (if already on conventional medications). 

#### 3.2.3. Theme 3: Challenges to Effective Diabetes Care Delivery

Overall, current health services provided for diabetes care in Nepal were described to have improved compared to the past. However, based on their experiences, HCPs reported a number of barriers to effective service delivery. 

##### **Sub-Theme 1**: Shortages of Quality Healthcare Facilities and Workforce

Whilst healthcare facilities and the workforce had improved with time, the improvements were reported to be mostly in the cities, particularly in Kathmandu, where there had been increases in qualified HCPs and healthcare facilities for diabetes management. Rural areas and areas surrounding Kathmandu Valley were reported to lack effective diabetes treatment and monitoring services. 

Participants further believed that the current healthcare services and facilities, as well as the workforce were still insufficient for efficient and effective handling of the increasing number of diabetes cases. Most participants believed that there was a lack of professionals who can educate patients, such as diabetes educators and diabetes nurses, ‘*there is not one diabetes educator in Nepal’* (P27, Physician), and *‘proper… or qualified pharmacists out here’* (P10, Physician) for effective medication counselling services. 

Others felt that there were inadequate numbers of podiatrists and dieticians. The need for, and lack of, effective services, including appropriately trained healthcare professionals, to promote physical activity in Nepal was emphasised. 

Similarly, whilst some reported that not all laboratory services could be trusted equally, a few were concerned about the lack of internationally recommended Diabetes Control and Complications Trial standard methods of monitoring HbA1c in the laboratories ‘*even in Kathmandu*’ (Table 4, Q1). Lack of infrastructure to ‘*maintain the cold-chain*’ for effective storage and preservation of insulin was reported as a challenge in remote areas (P12, Physician). 

Participants felt that the healthcare system and healthcare policies were guided by *‘adhocism’* (P25, Ayurvedic physician), without any evidence-based or long-term planning, both in terms of the care of patients with diabetes, and in preventative care. They felt that the overall approach to diabetes care lacked ‘*sensitivity*’ towards patients’ needs and that the Nepalese healthcare system did not have resources to identify vulnerable patients, or those who were not receiving appropriate treatment and were at risk because of uncontrolled blood glucose levels.

##### **Sub-Theme 2**: Daily Burden of Patient Care

The physicians reported that they faced a large daily patient burden. Participants in government hospitals revealed that in addition to the patients from Kathmandu, they also received a large number of rural patients and patients from around Kathmandu Valley. Participants strongly believed that the burden was primarily caused by HCP shortages, and patients’ desire to seek treatment from ‘well-known’ physicians. The high patient burden and the need to allocate sufficient consultation time to each patient was noted as a constant challenge compromising the level of diabetes care. 

##### **Sub-Theme 3**: Inconsistency in Care Provision

Participants believed that physicians provided varying levels of care to patients, particularly in the pharmacological treatments provided, and when treatment was commenced. Notably, participants felt that their rural counterparts did not provide an ‘*aggressive*’ initial treatment, which they believed avoided the ‘*legacy effect*’ in patients (P10, Physician). The varying levels of care were understood to be a reason for increasing ‘doctor shopping’ and impacted on patients’ confidence in prescribers. Lack of a consistent *‘line of treatment’* was also reported as a *‘drawback’* in Ayurvedic treatment (P2, Ayurvedic physician). Nonetheless, participants had noticed improvements in care, and *‘even the prescriptions from doctors who practice in the periphery’* (P3, Physician) were reported to have improved overtime. 

##### **Sub-Theme 4**: Lack of Effective Interprofessional Collaboration 

The majority of participants reported a lack of effective interprofessional collaboration in diabetes care, which they felt compromised effective diabetes management. There were only a few hospitals and clinics where nurses, dieticians, physicians and endocrinologists collaborated to deliver diabetes care. This collaborative care consisted of referrals by physicians for additional consultations with nurses and dieticians. In the majority of cases, overall diabetes care involved physician–patient consultations only, followed by patients visiting pharmacies for their medications (Figure 1). Furthermore, physicians were either the primary healthcare professionals delivering care directly to their patients or care was delivered by other healthcare professionals under physician supervision.

A key factor that negatively impacted collaboration was the perceived lack of support among HCPs. This was noted across the board, with physicians perceiving a ‘*lack of support*’ (P10, Physician) from pharmacists; pharmacists reporting lack of respect from physicians; and dieticians believing that some physicians would not refer patients to them. A few pharmacists and dieticians felt that if physicians encouraged their patients to visit them, it would not only promote their roles, but also facilitate patients’ dietary or medication management.

Participating pharmacists felt that doctors’ recognition of the role of a pharmacist and referral of their patients for consultation to pharmacists, could help generate awareness and make the public appreciative of the role of the pharmacist and pharmacy staff. 

#### 3.2.4. Theme 4: Perceived Strategies to Optimise Care in the Healthcare System

Several solutions were identified, ranging from training HCPs, to government policy changes. Participants felt that trained diabetes educators who could contribute effectively to diabetes care and reduce the burden on existing physicians were needed. This would allow physicians sufficient time to focus on treatment and management. 

Inter-professional collaboration, facilitated by mutual professional respect and recognition, was perceived as important for effective diabetes management. This was highlighted to effectively and more efficiently deal with the increasing numbers of diabetes cases in Nepal (Table 4, Q2). 

Developing and implementing guidelines for diabetes management was perceived as essential, particularly to address inconsistency in diabetes care (Table 4, Q3). While country specific guidelines were reported as necessary, participants believed that in order to develop guidelines specific to Nepal, it was imperative that epidemiological data were first collected instead of relying on ‘*extrapolated data*’ from other countries (Table 4, Q4). Similarly, research in Ayurvedic treatment modalities was considered essential to generate and disseminate information about their efficacy in diabetes management, and to upgrade the status of traditional medicines from being *‘time-tested’* to being *‘evidence based’* (P2, Ayurvedic physician). 

An integrative medicine approach was perceived as a solution to address patients’ desire for treatment with ‘natural’ (Ayurvedic) treatments without jeopardizing patients’ health because of the lack of effective diabetes control. Participating physicians, particularly those practicing traditional medicine felt that bringing the Ayurvedic treatment facility under one umbrella with modern medicine would not only provide options for patients and address their right to choose, but would be an effective approach to utilise the ‘best’ in each system. 

Some participants emphasised the need for responsible and ethical practice from all HCPs involved in diabetes care, and systems to regulate poor practice. 

A majority of participants reported that government initiatives, including adequate budget allocation and policies, should focus on non-communicable diseases, both in general and specifically on diabetes. This was considered a pre-requisite for implementing strategies to improve diabetes. The participants felt that there was a need for a strict implementation of regulations to ensure hospitals and HCPs acted in the best interests of the patients. They also believed that the government should address patients’ desire for traditional treatment methods by protecting and promoting Ayurvedic services. 

## 4. Discussion

Overall, this study demonstrated that healthcare service delivery for patients with T2D constituted information provision and education, together with treatment delivery through physician–patient consultations in most hospitals and clinics. Some additional diabetes-specific services, such as diabetes education classes and consultations with dieticians and nurses, were available in a few hospitals and clinics. Whilst physicians, dieticians, nurses and pharmacy staff were the main HCPs providing healthcare and medication-related services to the patients, as in many countries, the primary HCP was the physician. Very little interprofessional collaboration was reported, where physicians tended to work as single practitioners to deliver diabetes care in most settings, and others worked in their own professional silos. The shortage of diabetes educators was identified as a major challenge in effective education dissemination and overall diabetes management. Similarly, the lack of collaborative efforts between the HCPs was recognised as a significant barrier to effective diabetes treatment outcomes. These findings are not unique to Nepal, and the limited inter-professional collaboration by a healthcare team in the management of patients with diabetes and other chronic conditions appear to be an issue that has been noted previously [33,34]. 

Diabetes care is complex and the care process requires that many issues beyond glycaemic control are addressed [35]. A comprehensive diabetes management plan from a physician-coordinated team, including a range of HCPs, such as, physicians, nurse practitioners, nurses, dietitians, pharmacists, and mental HCPs with expertise and a special interest in diabetes is considered vital [4,35]. Whilst provision of care in hospitals providing diabetes specific services has demonstrated that there are emerging efforts in collaboration between physicians, nurses and dieticians in diabetes care in Nepal, the lack of collaboration between care providers, both within and across healthcare specialties and paradigms of care (traditional and modern methods of treatment) has appeared as a major barrier to diabetes care in Nepal. This is also an issue in other countries as patients seek treatments through both traditional and modern medicine. 

Multi-professional collaboration efforts in diabetes care in Nepal could not only improve diabetes management, but could help reduce the burden for physicians, decrease patients’ dependency on physicians for information and open opportunities for the ‘under-utilised’ professions, such as pharmacists and dieticians. Pharmacy staff in Nepal, in general, are playing, a rather passive role in diabetes management, primarily dispensing and offering brief counselling. In recent years, pharmacy globally has moved from being a product-centred profession to a profession involved in the delivery of health services tailored to the patients’ needs. Particularly in developed countries, adequately trained and competent pharmacists are delivering health promotion activities and interventions to facilitate diabetes management [36,37]. As Nepal faces a dearth of healthcare workforce [38], it is necessary that efforts are directed towards building the capacity of the existing healthcare workforce through training, recognition of roles and collaboration. Training pharmacy staff to be competent diabetes care providers and including them in diabetes care teams can be expected to aid diabetes management and improve outcomes [39,40]. A chronic care model is being employed for diabetes care in primary care settings in developed countries, such as the United States [41]. Trained and qualified pharmacist staff can run a diabetes management program through community pharmacies, with established mechanisms for referral to the physicians and other team members through chronic care models. Lack of trust in services delivered through pharmacies and by the pharmacy staff appears as a major barrier to incorporation of pharmacists/pharmacy staff in diabetes care in Nepal and is a key factor hindering the development of the role of pharmacies in patient care. To address this, it is important that active steps are taken to strengthen pharmacy services. 

Equally important and urgent in diabetes care in Nepal is the need to address the reported lack of diabetes educators. Education is the cornerstone of diabetes management [42]. However, educating and imparting knowledge to patients is a complicated process and requires considerable amount of time [43]. In Nepal, education-related concerns are complex and important because of the diversity in patient education and the general literacy level [44], and inadequate community awareness about diabetes [45]. Professionals are needed to provide structured diabetes education to patients and to assist in generating diabetes awareness. To date, there are no national ‘Diabetes Educator Certification’ provisions in Nepal, as in other countries; and as a result, there are not many diabetes educators. There are a few who have obtained certification internationally. There has been an increasing effort to train healthcare professionals, such as nurses [46] and community health volunteers [47] in diabetes education and management. In the settings where our interviews took place, nurses were mostly involved in diabetes education. The need for diabetes educators may be addressed by expanding these trainings to other HCPs (dieticians and pharmacists) [43] or by introducing new programs to generate qualified diabetes educators. The former could address the urgent need, while new training programs should be implemented to address the issue long term. 

In addition to the need for inter-professional collaboration and increased roles responsibility for healthcare professionals other than physicians, an integrative medicine approach was advocated by the participants to offer patients with options and to promote collaboration across paradigms (modern and traditional). While inter-professional collaboration involves integration within the general paradigm of biomedicine, integrative medicine involves integration across paradigms [48]. Integrative medicine is recognised and practiced in countries, such as Canada and the United States [48]. Conceptually both inter-professional collaboration and integrative medicine appears useful. Nepalese patients’ desire to be treated with ‘natural products/medicines’ compared to conventional (modern) medications has been previously identified [12]. Patients in Nepal often opt for natural medicines, delaying or discontinuing prescribed conventional medications [24]. An integrative-medicine approach, could therefore be expected to promote consistency in care, and to address the concerns observed amongst patients [12] in choosing traditional or modern medicines. 

Lack of adequate healthcare resources in rural areas in Nepal and the need to address this has been previously reported [10,49] and was also highlighted in this study. The difference in healthcare facilities and workforce between rural and urban areas can impact diabetes care in multiple ways. First, lack of easily accessible healthcare services can have a direct effect on patients’ health-seeking behaviour [50] which could create a delay in diagnosis and treatment [51]. Second, this compels patients to travel to cities for healthcare, increasing their healthcare costs. Third, it also adds to the ‘patient-load’ for HCPs in the cities and consequently impacts the quality of care delivered. Furthermore, the limited healthcare infrastructure limits the choices available for HCPs in rural regions to effectively manage diabetes. 

In Nepal, it is also important to consider that despite an increase in chronic conditions like diabetes, control of communicable diseases and issues related to maternal and child health [52,53] still take precedence in rural areas. Consequently, the skills (and priority) of the HCPs in rural areas may not be specifically suited to diabetes care. Therefore, whilst increasing healthcare facilities and workforce in rural areas is vital, equally important is to ensure that the HCPs in rural areas are effectively equipped and trained to manage diabetes. 

This study also identified lack of national guidelines and availability or accessibility of effective healthcare services to patients in rural areas as barriers to diabetes care in Nepal. This corroborates with previous research [10,54]. However, it highlights the need for a country such as Nepal, to develop and implement national guidelines that support effective diabetes care through an inter-professional and collaborative team of healthcare providers, who are respectful of each other, and willing to share the patient care management load. 

Although a part of the healthcare system, HCPs are significantly affected by the broader healthcare system (structures, facilities and manpower) and policies (or lack thereof); and collectively they influence diabetes management. The strategies and interventions that aim to improve diabetes management in Nepal need to carefully consider the deficiencies at the healthcare system level as well as at individual HCP level, both collectively as well as independently.

### Study Limitations

This study has two potential limitations. First, data collection was conducted within Kathmandu Valley, therefore, it cannot be generalised to other parts of the country, particularly to rural areas, where the healthcare setting is different to urban settings. Second, (certified) diabetes educators could not be recruited, and the study may have missed some important aspects pertaining to the opinions and experiences of diabetes educators. 

## 5. Conclusions

Existing healthcare services available to patients with T2D in Nepal were reported as being limited to physician–patient consultations in most cases. Few hospitals and clinics offered diabetes-specific services involving multiple HCPs. An inter-professional collaborative approach to diabetes care was lacking. Pharmacists and pharmacy staff operated in silos.

Improving outcomes for patients with chronic diseases such as T2D should be a healthcare system priority. Diabetes management in Nepal must shift from its current situation which focuses on episodic care to a comprehensive model of integrative medical care to achieve effective diabetes outcomes. 

## Figures and Tables

**Table 1:** Interview questions.

Key Broad Questions Asked in the In-Depth Interviews:	
1.	Could you please describe your typical role in providing services to patients with type 2 diabetes?
2.	What are the current healthcare services that are provided to patients with type 2 diabetes?
3.	How do you think these existing healthcare services influence patients’ diabetes care?
4.	What impact (of these services) have you seen?
5.	What strategies do you use to promote effective diabetes management?
6.	How do you think we can help patients better manage their diabetes overall?

**Table 2:** Demographic characteristics of the participants (N = 30).

Demographic Variable	N
**Gender**	
Male	19
Female	11
**Age Bracket**	
20–30 years	6
31–40 years	13
41–50 years	5
51–60 years	4
61–70 years	2
**Profession**	
Physicians (General Physicians, Internal Medicine specialists, Endocrinologists, Diabetologists and Sports Physician)	13
Ayurvedic physicians *	6
Pharmacy staff	5
Pharmacists (Hospital + Community)	3 (2 + 1)
Pharmacy Assistant ** (Community)	1
Health assistant *** (Community)	1
Nurses	2
Dieticians	4
**Professional Experience (years)**	
Range	0.5–36
Median	8
Mean	13.1
**Approximate Number of Patients with Type 2 Diabetes Served/Week**	
<5	1
5–10	4
11–19	9
>20	16

* Physicians of Ayurveda and traditional medicines; ** Pharmacy staff with Intermediate in Pharmacy; *** Pharmacy staff without a formal education in pharmacy but education in general health with a brief training on medication dispensing.

**Table 3:** Interpreted themes.

Themes
Delivering care for patients with diabetes
2. Healthcare professionals partnering for wholistic care in diabetes management
3. Challenges to effective diabetes care delivery 4. Perceived strategies to optimise care in the healthcare system

**Table 4:** Representative quotes.

Quotes Illustrating the Problems in Healthcare Provision and Their Solutions	
**Q1**	Deficiency in monitoring *‘The problem with HbA1c (monitoring) in Nepal is, standard kits are not being used. When standard kits are not used, then, despite paying so much money, you are not getting a good reliable report. That is also a big problem here. Although they claim it! … So, meaning, internationally recommended processes, methods are not used, no?’* (P14, Physician)
**Q2**	Collaboration and trainings to build qualified manpower *‘We need to develop a team. No, we should not go only for physicians, there has to be nursing … educators… means anybody who can convincingly take classes like it could be social-worker or teacher or volunteer, no? Who can…where we educate them about diabetes and they will use the teaching material to teach the local people about diabetes and its prevention, and the patients about diabetes and its control…and its monitoring, no? So, from what I have observed in Nepal is, we don’t have a team. We need training facilities (to develop) diabetes educator, and we need nurses working in diabetes to help, an…in you know checking and monitoring them, and then we need, an… people physicians with special interest in diabetes! Of course, diabetes should be treated by any physician, but diabetes has progressed so rapidly in its treatment and its monitoring, no? That now you cannot (easily keep up). So, we need people who are really interested and educated about diabetes treatment’.* (P14, Physician)
**Q3**	Inconsistency in care and need for guidelines ‘ *An individual’s effort is not sufficient. For example, if I say, if I prescribe (a medication), saying that “you have to take it”. Now he will go to the other doctor. Why? (because the patient thinks, the next doctor) may be he will say “don’t take (medications)?” Now this next doctor has to say “you have to take the medications”. Here, even between doctors this doesn’t match. Now when here I have said that “you have to take it”, then he (the other doctor) says… “no, no..” to show that he is better than the others… “don’t, don’t take the medicines now… you increase the Ayurvedic, exercise… and then come after 3 months”, for the patient, that (doctor) is good. For patients, doctor who does prescribe insulin or medicines… that doctor is good! We have to change this. What I write should not be contradicted by the other!… The patient may visit five doctors… doctor shop…if all five voice the same, that “you need medicines”, only then patients will accept, “medicines are really required for me”*! *So we should have this system in Nepal. There has to be one guideline… that tells that when the sugar level reaches this and this, you should take medications. The guidelines which all the doctors will follow..then, whichever doctor you go to, it will be the same*’ (P18, Physician)
**Q4**	Need for national data/ research *‘(Necessary is) Data! Data collection, no? Anybody in the world asks me what is the situation of diabetes in Nepal. I feel like, “hmm… in my clinic I see a 100 patient…” and “what happen to them?”, “I don’t know”. So…ya... that’s like the saddest part! That we have no data collection, we have no epidemiology. Everything is in pockets. There is no national centre. We don’t know how many of these are ending up with cancer, what is happening with the thyroid patients… there is like no data. And that is what is really scary. Just working in the dark. Thinking they are right… extrapolating data from India, or China or America and then you are just putting them onto your people, which has been time and again proved that that is not the same.’* (P27, Physician)
